# Angiogenic Potential of Tissue Engineered Cartilage From Human Mesenchymal Stem Cells Is Modulated by Indian Hedgehog and Serpin E1

**DOI:** 10.3389/fbioe.2020.00327

**Published:** 2020-04-17

**Authors:** Yannick Nossin, Eric Farrell, Wendy J. L. M. Koevoet, Rodrigo A. Somoza, Arnold I. Caplan, Bent Brachvogel, Gerjo J. V. M. van Osch

**Affiliations:** ^1^Department of Otorhinolaryngology, Head and Neck Surgery, Erasmus MC, University Medical Center, Rotterdam, Netherlands; ^2^Department of Oral and Maxillofacial Surgery, Erasmus MC, University Medical Center, Rotterdam, Netherlands; ^3^Department of Biology, Skeletal Research Center, Case Western Reserve University, Cleveland, OH, United States; ^4^Center for Multimodal Evaluation of Engineered-Cartilage, Case Western Reserve University, Cleveland, OH, United States; ^5^Department of Pediatrics and Adolescent Medicine, Experimental Neonatology, Faculty of Medicine, University of Cologne, Cologne, Germany; ^6^Faculty of Medicine, Center for Biochemistry, University of Cologne, Cologne, Germany; ^7^Department of Orthopedics, Erasmus MC, University Medical Center, Rotterdam, Netherlands

**Keywords:** angiogenesis, microarray, SerpinE1, IHH, VEGFa, BMSC, chondrogenesis, secretome

## Abstract

With rising demand for cartilage tissue repair and replacement, the differentiation of mesenchymal stem cells (BMSCs) into cartilage tissue forming cells provides a promising solution. Often, the BMSC-derived cartilage does not remain stable and continues maturing to bone through the process of endochondral ossification *in vivo*. Similar to the growth plate, invasion of blood vessels is an early hallmark of endochondral ossification and a necessary step for completion of ossification. This invasion originates from preexisting vessels that expand via angiogenesis, induced by secreted factors produced by the cartilage graft. In this study, we aimed to identify factors secreted by chondrogenically differentiated bone marrow-derived human BMSCs to modulate angiogenesis. The secretome of chondrogenic pellets at day 21 of the differentiation program was collected and tested for angiogenic capacity using *in vitro* endothelial migration and proliferation assays as well as the chick chorioallantoic membrane (CAM) assay. Taken together, these assays confirmed the pro-angiogenic potential of the secretome. Putative secreted angiogenic factors present in this medium were identified by comparative global transcriptome analysis between murine growth plate cartilage, human chondrogenic BMSC pellets and human neonatal articular cartilage. We then verified by PCR eight candidate angiogenesis modulating factors secreted by differentiated BMSCs. Among those, Serpin E1 and Indian Hedgehog (IHH) had a higher level of expression in BMSC-derived cartilage compared to articular chondrocyte derived cartilage. To understand the role of these factors in the pro-angiogenic secretome, we used neutralizing antibodies to functionally block them in the conditioned medium. Here, we observed a 1.4-fold increase of endothelial cell proliferation when blocking IHH and 1.5-fold by Serpin E1 blocking compared to unblocked control conditioned medium. Furthermore, endothelial migration was increased 1.9-fold by Serpin E1 blocking and 2.7-fold by IHH blocking. This suggests that the pro-angiogenic potential of chondrogenically differentiated BMSC secretome could be further augmented through inhibition of specific factors such as IHH and Serpin E1 identified as anti-angiogenic factors.

## Introduction

Mesenchymal Stem Cells (BMSCs) are multipotent progenitor cells that can be isolated from a large variety of tissues such as bone marrow, synovium, adipose tissue and umbilical cord and can be differentiated *in vitro* toward the chondrogenic lineage (Pittenger et al., 1999; Somoza et al., 2014). Expression of Collagen Type X and Alkaline Phosphatase show a chondrocyte phenotype that resembles that of the chondrocytes found in the hypertrophic zone in the *in vivo* growth plate (Yoo et al., 1998; Zimmermann et al., 2008; Hellingman et al., 2010; Farrell et al., 2011) during endochondral ossification. Moreover, BMSC-derived cartilage constructs that are implanted subcutaneously in mice or rat, promote the transition of cartilage to bone via the invasion of blood vessels into the constructs (Pelttari et al., 2006; Cui et al., 2007; Scotti et al., 2010; Marino, 2011; Staines et al., 2013; Walzer et al., 2014; Thompson et al., 2015). This is driven by the formation of new vessels from preexisting vessels (known as angiogenesis), which is mainly induced and directed by secreted factors (Otrock et al., 2007; Rocha et al., 2014). Soluble factors secreted by BMSC-derived cartilage are proposed to have a pro-angiogenic capacity (Rocha et al., 2014) by stimulating the proliferation of endothelial cells and their migration into the cartilage template (Otrock et al., 2007) to promote subsequent vessel formation. This process requires a finely tuned interplay between pro- and anti-angiogenic factors to form fully functional vessels (Iruela-Arispe and Dvorak, 1997).

In this study, we identified soluble factors in the secretome of chondrogenically differentiated bone marrow-derived BMSCs that can modulate angiogenesis. We first confirmed the effect of the secretome of chondrogenically differentiated BMSCs on angiogenic capacity using a set of different angiogenesis assays: the chicken chorioallantoic membrane assay (CAM) and commonly used *in vitro* assays for migration and proliferation using Human Umbilical Vein Endothelial Cells (HUVEC). We then used global transcriptome comparison of existing data sets from murine growth plate cartilage (Iruela-Arispe and Dvorak, 1997), healthy human articular cartilage and healthy human chondrogenic BMSCs (Somoza et al., 2018) to identify expressed factors which may be secreted by chondrogenic BMSC constructs to mediate angiogenic effects in these assays. Finally, we studied the role of these factors in CAM and HUVEC proliferation and migration assays by applying neutralizing antibodies. Here, we show that IHH and Serpin E1 act as anti-angiogenic factors, as they are secreted by chondrogenically differentiated BMSCs and prevent endothelial cell proliferation and migration into BMSC derived cartilage constructs.

## Materials and Methods

### Chondrogenic Differentiation of BMSCs and Generation of Conditioned Medium

Mesenchymal stem cells were isolated from seven human bone marrow samples aspirated from patients undergoing total hip arthroplasty after informed consent (MEC-2004-142 and MEC-2015-644). In total, seven donors were used, 4 female and 3 male (age range from 20 to 63–71) were used. Cells were plated at a density of 2,300 cells/cm^2^ in expansion medium, α-MEM (Gibco, Dublin, Ireland) containing 10% FCS (Gibco, Basel, Switzerland), supplemented with 1 ng/mL FGF2 (BioRad, Hercules, CA, United States), 10 mM ascorbic acid-2-phosphate (Fluka, Charlotte, NC, United States), 1.5 μg/mL fungizone (Gibco) and 50 μg/mL gentamicin (Gibco) at 37°C and 5% CO_2_). After 24 h, non-adherent cells were removed and adherent cells were expanded in the above-mentioned medium. At confluence, cells were passaged, and seeded at 2,300 cells/cm^2^. For generation of pellets, BMSCs at passage three were utilized (≈3 population doublings in passage 2 and 3 population doublings in passage 3. Population doublings in passage 1 are unknown as we do not know the exact number of MSCs in the fresh bone marrow biopsy).

Cartilage was obtained from seven patients (4 males, 3 females, ages between 63 and 86) undergoing total knee replacement surgery for osteoarthritis with implicit consent of the use of leftover material after surgery (after approval by the local ethics committee; MEC-2004-322). Full thickness cartilage was harvested, treated with 2 mg/mL protease in physiological saline solution (Sigma-Aldrich, St. Louis, MO, United States) for 90 min and subsequently digested overnight in basal medium [DMEM, 4.5 g/L glucose with 10% fetal calf serum (FCS), 50 μg/mL gentamicin, and 1.5 μg/mL fungizone (all Invitrogen, Carlsbad, CA, United States)] supplemented with 0.12 U collagenase B (Roche Diagnostics, Almere, the Netherlands). The next day, the resulting cell number was determined using a hemocytometer. The primary chondrocytes were then seeded at a density of 7,500 cell/cm^2^ in T175 culture flasks for expansion with the above-mentioned basal medium. For generation of stable cartilage pellets, chondrocytes at passage 1 were utilized.

Pellet cultures of BMSCs and primary chondrocytes were formed by seeding 2.0 × 10^5^ cells in 0.5 mL in a 15 mL conical polypropylene tube and centrifuging for 8 min at 300 g. Pellets from both cell sources were cultured for 21 days in chondrogenic medium, high-glucose DMEM supplemented with 50 μg/mL gentamicin (Invitrogen), 1.5 μg/mL fungizone (Invitrogen), 1 mM sodium pyruvate (Invitrogen), 40 μg/mL proline (Sigma, Kawasaki, Kanagawa Prefecture, Japan), 1:100 v/v insulin-transferrin-selenium (ITS; BD Biosciences, San Jose, CA, United States), 10 ng/mL Transforming Growth Factor β1 (R&D Systems), 10 mM ascorbic acid-2-phosphate (Sigma), and 100 nM dexamethasone (Sigma). The medium was renewed twice a week. At day 21, medium was renewed and 24 h later the pellets were washed three times with PBS and then incubated with basal medium (UCM) consisting of phenol-red free DMEM (Gibco) supplemented with 0.1% w/v BSA (Sigma) and 10 mM ascorbic acid-2-phosphate (Fluka) for 24 h to produce conditioned medium (CM) for downstream experiments. This CM was collected, cell debris removed by centrifugation at 300xg for 8 min and stored at –80°C. Pellets were digested in 350 μL RNABee (Tel-Test. Inc., Pearland, TX, United States) and stored at –80°C for subsequent RNA-isolation, cDNA synthesis and gene expression analysis. In addition, pellets were fixed in 4% formalin at room temperature overnight and then processed for histological analysis.

### Gene Expression Analysis

To isolate RNA, the pellets in RNABee were homogenized with an Eppendorf- Micro-pestle (Eppendorf, Hamburg, Germany). Total RNA isolation was performed according to manufacturer’s protocol utilizing the RNeasy Column system (Quiagen, Hilden, Germany). The RNA concentration was determined using a NanoDrop^®^ spectrophotometer (Isogen Life Science, Utrecht, the Netherlands). 0.5 μg RNA was used for cDNA synthesis following the protocol of the manufacturer of the RevertAid First Strand cDNA kit (Thermo Fisher Scientific, Waltham, MA, United States). Gene expression was analyzed by real-time Reverse Transcription Quantitative Polymerase Chain Reaction (RT-qPCR) on a StepOnePlus^TM^ System using SYBR Green (Applied Biosystems, Foster City, CA, United States) or Taqman (Thermo Fisher Scientific, Waltham, MA, United States) assays. Primers for Syber Green RT-qPCR analysis *(ENPP2, IHH, NDNF, RAMP1, SRPX1, ADM, SERPIN E1*) were purchased as assays-on-demand from BioRad. Sequences of additional primers and probes for Taqman and Syber PCR analysis are Alkaline Phosphatase, Biomineralization Associated (*ALPL) (Fw:* GGC AATAGCAGGTTCACGTACA; Rev: CGATAACAGTCTTGC CCCACTT; Probe: CCGGTATGTTTCGTGCAGCCATCCT); Collagen Type II Alpha 1 Chain (COL2A1) (Fw: GGCAAT AGCAGGTTCACGTACA; Rev: CGATAACAGTCTTGC CCCACTT; Probe: CCGGTATGTTTCGTGCAGCCATCCT); Collagen Type X Alpha 1 Chain (COL10A1) (Fw; CAAGG CACCATCTCCAGGAA; Rev: AAAGGGTATTTGTGGC AGCATATT; Probe: TCCAGCACGCAGAATCCATCTGA); Glyceraldehyde-3-Phosphate Dehydrogenase (GAPDH) (Fw: GTCAACGGATTTGGTCGTATTGGG; Rev: TGCCATGGGT GGAATCATATTGG; Probe: TGGCGCCCCAACCAGCC); Hypoxanthine Phosphoribosyltransferase 1 (HPRT1) (Fw: TAT GGACAGGACTGAACGTCTTG; Rev: CACACAGAGGGC TACAATGTG; Probe: AGATGTGATGAAGGAGATGGGAG GCCA); Ribosomal Protein S27 (RPS27) (Fw: TGGCTGTCCT GAAATATTATAAGGT; Rev: CCCCAGCACCACATTCATCA); Vascular Endothelial Growth Factor A (VEGFA) (Fw: CTT GCCTTGCTGCTCTACC; Rev: CACACAGGATGGCTTG AAG). The best housekeeper index (BKI) was calculated from *GPDH, RSP27* and *HPRT* and used for the 2^–ΔCT^ method.

### Histology

Fixed pellets were embedded in 1% agarose and then further in paraffin using standard procedures. Six μm sections were cut and further processed by deparaffinizing and rehydrating. At least three pellets per condition were sectioned.

#### Glycosaminoglycan Staining

Deposition of glycosaminoglycan (GAG) was determined by thionine staining. Paraffin sections were stained for 5 min in 0.4% thionine in 0.01 M of aqueous sodium acetate, resulting in a selective staining for glycosaminoglycans (Bulstra et al., 1993).

#### Immunohistochemical Staining for Collagen Type II and Collagen Type X

Following deparaffinization and rehydration, sections were treated with 1% w/v hyaluronidase to increase antibody penetration and antigen retrieval was performed using 0.1% w/v pronase, both were sequentially incubated for 30 min at 37°C. Following this, the sections were blocked using normal goat serum (Southern Biotech, Birmingham, AL, United States). Sections were incubated with either mouse monoclonal antibody against collagen type II 0.4 μg/mL (Developmental Studies Hybridoma Bank, Cat.# II-II6B3) for 60 min or collagen type X 5 μg/mL (Thermo Fisher Scientific, Clone X53, Cat.# 14-9771-82) in PBS 1%BSA overnight. Both were incubated with a biotinylated goat anti-mouse antibody for 30 min followed by an incubation with ALP-conjugated streptavidin. Staining was revealed by incubation with a New Fuchsin substrate (Chroma, Kongen, Germany). Corresponding isotype controls for both antibodies were used (0.4 and 5 μg/mL of an isotype immunoglobulin G1 monoclonal antibody, respectively).

### Global Gene Expression Analysis

#### Candidate Angiogenesis Modulating Factor Selection From Previously Published Microarrays

The aim of this analysis was to identify known secreted angiogenic factors released from chondrogenic pellets. We first compared data from different zones of the murine growth plate (proliferative, pre-hypertrophic and hypertrophic) via paired *t*-test and selected factors with a cut off value of 3-fold change in the expression of genes. The selected list of uniquely expressed factors where then overlapped with different online resources from Uniprot, Geneontology and the Matrisome Project (Hynes and Naba, 2012) utilizing the Funrich software^1^ (Pathan et al., 2015) to select for secreted genes associated with the regulation of angiogenesis. The selected murine genes were then used as input for comparison with a human microarray to select those genes differentially expressed between chondrogenically differentiated BMSCs and newly formed articular cartilage with a criterion of either being expressed in one condition and not the other or having a 3-fold difference.

For comparison of the different growth plate zones, we used a dataset previously generated and described by Belluoccio et al. (2010). In brief, femoral growth plates were isolated from long bones of two 14 days old female Swiss White mouse. Using microdissection of frozen sections, ≈2,000 chondrocytes (per layer) were isolated from the proliferative (PR), pre-hypertrophic (PH) and hypertrophic (H) layer of the growth plate. Total RNA was extracted using PicoPure RNA isolation kit (Arcturus Bioscience, San Diego, CA, United States), treated with DNase, linearly amplified using MessageAmp aRNA kit (Thermo Fisher Scientific) labeled with Cy3/Cy5 fluorophores, then hybridized to 44k whole genome oligo microarrays (G4122A; Agilent Technologies, Santa Clara, CA, United States) and scanned on an Axon 4000B scanner. Features were extracted using GenePix Pro software (version 4.1; Axon Instruments, San Jose, CA, United States). The microarray data have been validated by qPCR on amplified RNA (Belluoccio et al., 2010).

To compare the murine data with a human model similar to the one used in this study, we overlapped the dataset with a list of genes we previously determined with microarray analysis. In this dataset, the transcriptome of human neonatal articular cartilage from femoral and tibial plateau of 1 month old cadaveric specimens (*n* = 2) was compared with chondrogenically differentiated BMSCs of two healthy adult volunteer donors (Somoza et al., 2018). In brief, RNA was isolated after homogenization with RNeasy mini columns (Quiagen), total RNA was linearly amplified and biotin labeled using Illumina TotalPrep^TM^ kits (Life Technologies) and whole-genome expression analysis was carried out using Illumina (CA) Human Ref-8v3 or Human HT-12 v4 BeadArrays^TM^. The cRNA was hybridized to Illumina BeadChips^TM^, processed and read using a BeadStation^TM^ array reader according to the manufacturer’s instructions (Illumina). Values of less than 130 relative fluorescence units were considered to be non-specific background signals.

### Angiogenesis Assays

Commercially derived pooled Human Umbilical Vein Endothelial Cell (HUVEC) (Lonza, Basel, Switzerland) were seeded at a density of 5 × 10^3^ cells/cm^2^ in culture flasks and cultured in endothelial growth medium (EGM-2 Promocell, Heidelberg, Germany). Medium was renewed every 2–3 days. When they neared confluency, cells were detached with 0.05% Trypsin-EDTA (Gibco) and used for angiogenesis assays. For angiogenesis assays, HUVECs between passages 8 and 10 were used.

#### Endothelial Cell Migration Assay

Migration assays were performed by seeding HUVEC (5 × 10^4^ cells/well) in 24-well Transwell inserts (8 μm pore size, Corning Life Sciences) in serum-free medium containing 0.05% BSA (Merk). The CM from chondrogenically differentiated BMSCs was placed in the lower compartment of different wells and diluted 1:1 with Endothelial Basal Medium (EBM-2, Promocell). EBM and non-conditioned medium are used as negative controls and Endothelial Growth Medium (EGM-2) (EBM-2 supplemented with EGF, FGF2, IGF-1, VEGFa, AA, Heparin, Hydrocortisone, and FBS) (Promocell) is used as positive control. After 10 h of incubation at 37°C 5% CO_2_, the cells on the membrane were fixed with 4% formaldehyde/PBS and the non-migrated cells from the upper surface of the membrane were removed with a cotton swab. We confirmed that no cell proliferation took place during the 10 h incubation, based on cell count. The migrated cells on the lower surface of the membrane were then stained with DAPI and then quantified by fluorescence microscopy and image analysis through ImageJ utilizing the included particle analysis macro. Five non-overlapping pictures were taken and analyzed per well for three independent experiments (Schindelin et al., 2012).

#### Endothelial Proliferation

To measure proliferation, 2.5 × 10^3^ HUVEC/cm^2^ were seeded in 48-well plates. After 24 h, medium was replaced with endothelial basal medium to synchronize the cells. After 8 h, cells were stimulated with either CM, EGM-2 as positive control or EBM-2 as negative control for 24 h. With the stimulus, 10 μM dEdU (BaseClick, Neuried, Germany) was added to stain DNA of replicating cells to allow assessment of proliferation. After 24 h, the cells were washed three times with phosphate-buffered saline and fixed with 4% formalin for 5 min. The EdU label was then revealed according the protocol provided by the manufacturer. The cells were counterstained with DAPI and imaged using fluorescence microscopy. Utilizing the particle analysis macro in ImageJ, we determined the amount of positively stained cells for DAPI and EdU and the percentage of cells positive for EdU was calculated.

#### Chick Chorioallantoic Membrane (CAM) Assay

Fertilized chicken eggs laid the day before (purchased from Drost Loosdrecht B.V.) were incubated sideways at 37°C and 65% humidity for 3 days before rupturing the air sack located at the blunt side of the egg, as well as opening a small hole on the top to deflate the air-sack and lower the fluid level. After 3 days of incubation, part of the shell was removed and filters containing concentrated CM were placed on top of the CAM. Therefore, CM harvested from chondrogenically differentiated BMSCs was concentrated utilizing the Amicon^®^ Ultra-2 mL Centrifugal Filters (Merck, Kenilworth, NJ, United States) and a sterile filter disks with a diameter of 5 mm were soaked with 5 μL of the concentrated CM and placed on the membrane. As a negative control, concentrated basal medium was utilized and 100 ng FGF2 was used as positive control (Schindelin et al., 2012). After 3 days of incubation, the CAM was fixed with 4% Formalin/PBS solution and the removed from the egg. Induction of vessel formation was assessed by taking pictures of each filter on the membrane, and the blinded pictures were ranked by three independent observers. A total of 45 images were ranked (45 being the highest and one the lowest rank). The inter-observer correlation was tested and the final rank determined by averaging the ranks per image.

### Antibody Blocking

To evaluate the effect of the selected factors, we neutralized their effect in CM by incubating with the corresponding antibodies for 30 min before utilization in the different assays. Blocking antibodies against VEGF-A (Cat. # AF-293-SP) and Serpin E1 (Cat.# MAB1786-SP, Clone # 242816) were obtained from R&D-Systems (Minneapolis, MN, United States) and utilized at 0.5 μg/mL according to the manufacturers *in vitro* testing protocol. The IHH neutralizing antibody (Cat.# 5E1) was purchased from DSHB (Iowa City, IA, United States) and used at 5 μg/mL according to the manufacturers specifications.

### Data Analysis and Statistics

The statistical analyses of gene expression data were performed on three replicates for each of the seven BMSC and seven chondrocyte donors. Since in some conditions certain genes were not expressed and a Shapiro-Wilk test revealed the absence of a normal distribution we chose a non-parametric analysis. Technical triplicates per donor were averaged and the differences between conditions were statistically tested by a Mann–Whitney *U*-test. The *in vitro* angiogenesis tests were performed in two batches of BMSC CM; for each batch, the media of three donors were pooled. These batches were tested in triplicate in independent experiments. Due to the continuous nature of the data it was depicted as a bar graph and for statistical evaluation of the data, a linear mixed model was utilized with a Bonferroni *post hoc* test. For the CAM assay, the inter-observer correlation was tested through a Spearman-correlation test. Per sample the rank of two observers was averaged and then per condition graphed as a boxplot with whiskers. The conditions were compared using the average rank of three observers with a Kruskal-Wallis one-way analysis of variance with ensuing Bonferroni *post hoc* test. For the *in vitro* and *in vivo assays* positive controls were included in the experiments to be able to exclude possible technical failures. These positive controls were omitted from statistical analyses as they are not biologically meaningful for the conclusions. Statistical analysis was performed using SPSS11 for Windows (IBM, Armonk, NY, United States).

## Results

### Conditioned Medium From Chondrogenically Differentiated BMSCs Induces Angiogenesis

**FIGURE 1:**
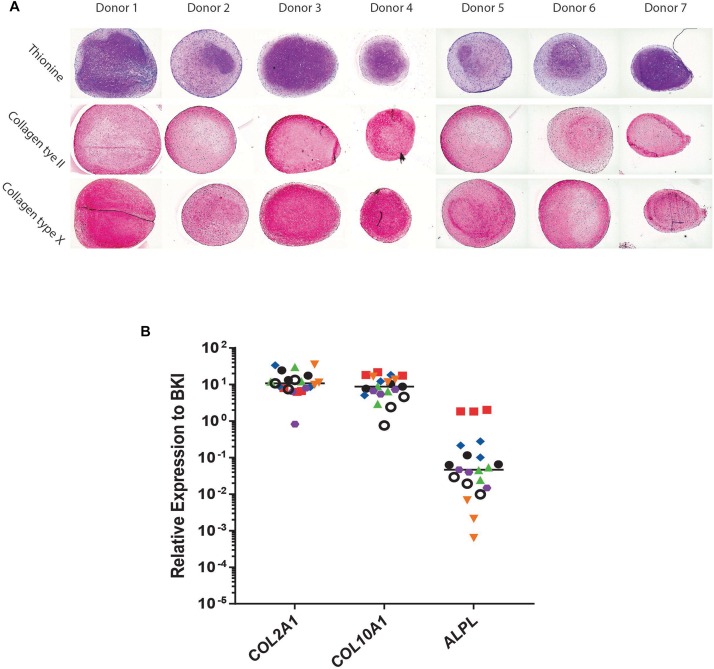
Chondrogenic differentiation of bone marrow derived mesenchymal stem cells (BMSC). **(A)** Histological and Immunohistological staining of day 21 chondrogenically differentiated BMSC-pellets of the donors for Collagen type II, Thionine and Collagen type X. **(B)** qPCR Gene-expression analysis of chondrocyte and hypertrophy marker genes in day 21 chondrogenically differentiated BMSCs. Raw average Ct value for COL2A1 = 20.54; COL10A1 = 20.99; ALPL = 28.19.

Conditioned medium was generated from day 21 chondrogenically differentiated BMSC-pellets and its angiogenic potential was evaluated. Cells of all donors used were successfully differentiated toward a chondrocyte-like phenotype as shown by thionine and type II collagen staining (Figure 1A). The BMSC-pellets also underwent hypertrophy as shown by deposition of type X collagen protein (Figure 1A). Further, gene expression analysis showed clear collagen type II, collagen type X and alkaline phosphatase expression in chondrogenic BMSC pellets.

**FIGURE 2:**
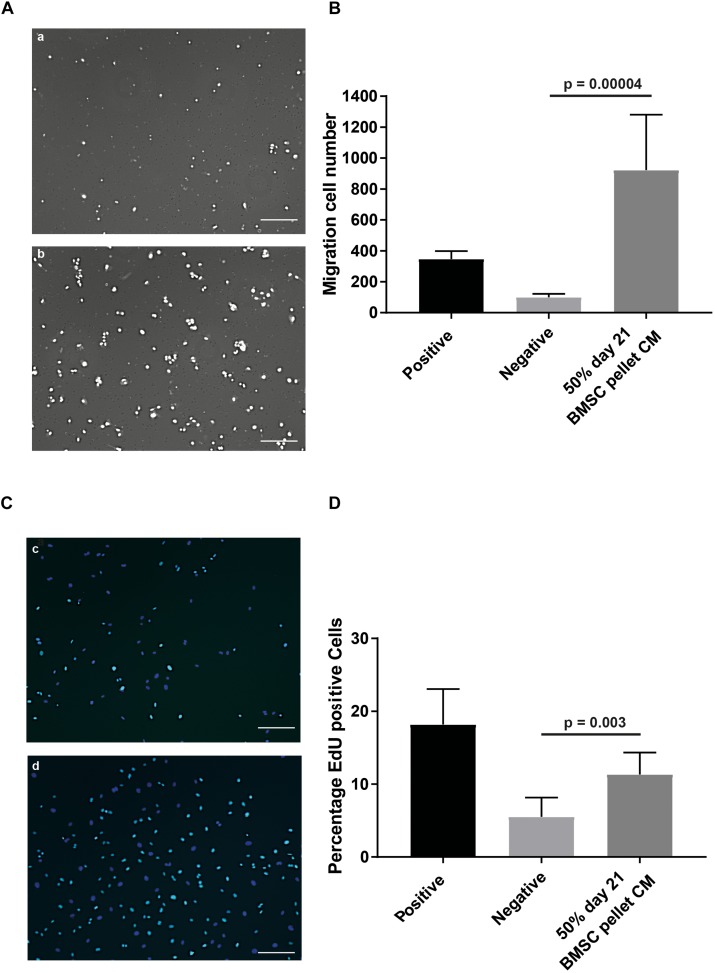
*In vitro* angiogenesis assays confirmed pro-angiogenic potential of CM of chondrogenically differentiated BMSCs in pellets. **(A)** Modified Boyden chamber endothelial migration assay images; (a) Negative control, 50% endothelial basal medium and 50% non-CM; (b) positive control, endothelial growth medium. **(B)** Quantification of amount of migrated cells stimulated by the CM. **(C)** Endothelial proliferation assay; (c) negative control; (d) positive control showing in cyan positive EdU staining, counterstained with DAPI (blue). **(D)** Quantification of difference in actively proliferating HUVECs after 24 h. Both experiments were performed in two batches each pooling 3 donors; *n* = 3. Scalebar = 200 μm, Statistical analyses were performed with mixed-linear model with Bonferroni *post hoc* test.

To assess the effects of CM on specific aspects of angiogenesis, we performed an endothelial cell migration assay utilizing a modified Boyden chamber assay. At a concentration of 50%, the CM induced a 9.2 (±4.5) fold increase (*p* = 0.00004) in the number of cells migrating compared to the non-CM (Figures 2A,B). To evaluate the effect of CM on endothelial cell proliferation the EdU incorporation in an *in vitro* HUVEC proliferation assay, was assessed. Exposure to CM resulted in 2.1 (±0.4) fold (*p* = 0.003) increase in EdU incorporation, indicating factors secreted by chondrogenically differentiated BMSCs increase endothelial cell proliferation (Figures 2C,D).

**FIGURE 3:**
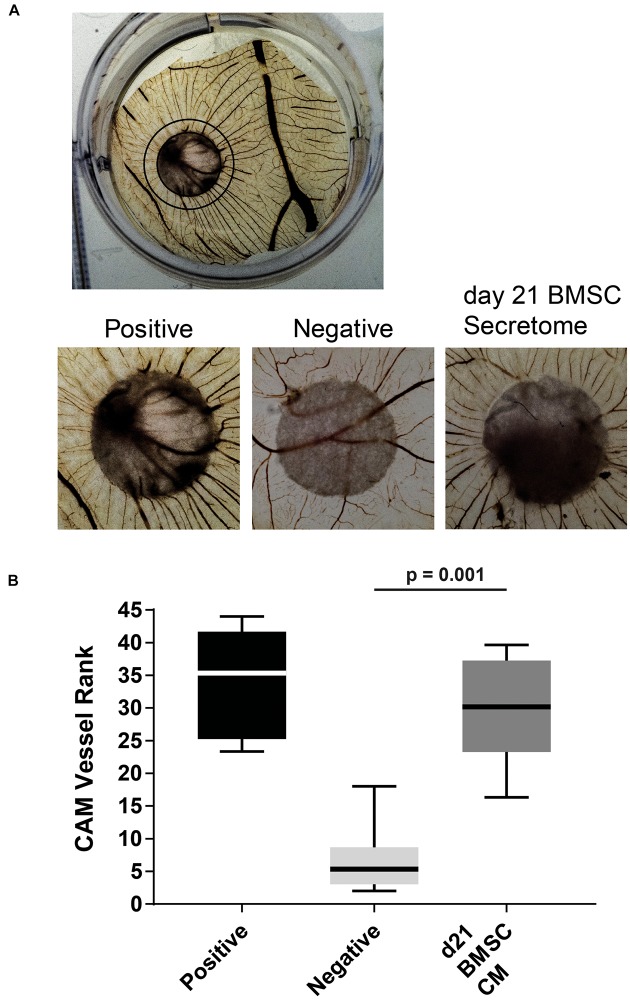
*In vivo* CAM assay illustrated the pro-angiogenic potential of chondrogenically differentiated BMSC secretome. **(A)** Overview of treated CAM [Black circle depicts approximate range of influence of placed filter (center)]. Magnifications of chick chorioallantoic membrane incubated for 72 h with CM soaked 5 mm filter disks. Filter circles were placed on a vessel free area on day 7 of the chick development. **(B)** Boxplot depicting the ranking of the angiogenic potential (lowest = 1 highest = 45: average of 3 independent observers; box = interquartile range, whiskers + 1–99%). *N* = 6 per condition.

Next, we tested the BMSC-derived CM in an *in vivo* chick chorioallantoic membrane (CAM) assay in which it induced an increase in angiogenesis compared to non-CM controls (Figure 3A). The observed vessels had a high degree of directionality toward the stimulus containing filter-disks as ranked by three independent observers (*p* = 0.001; Figure 3B). These results confirm that the secretome of chondrogenically differentiated BMSCs can induce angiogenesis.

### Selection of Eight Candidate Secreted Angiogenesis Regulating Factors From Microarray Datasets

**FIGURE 4:**
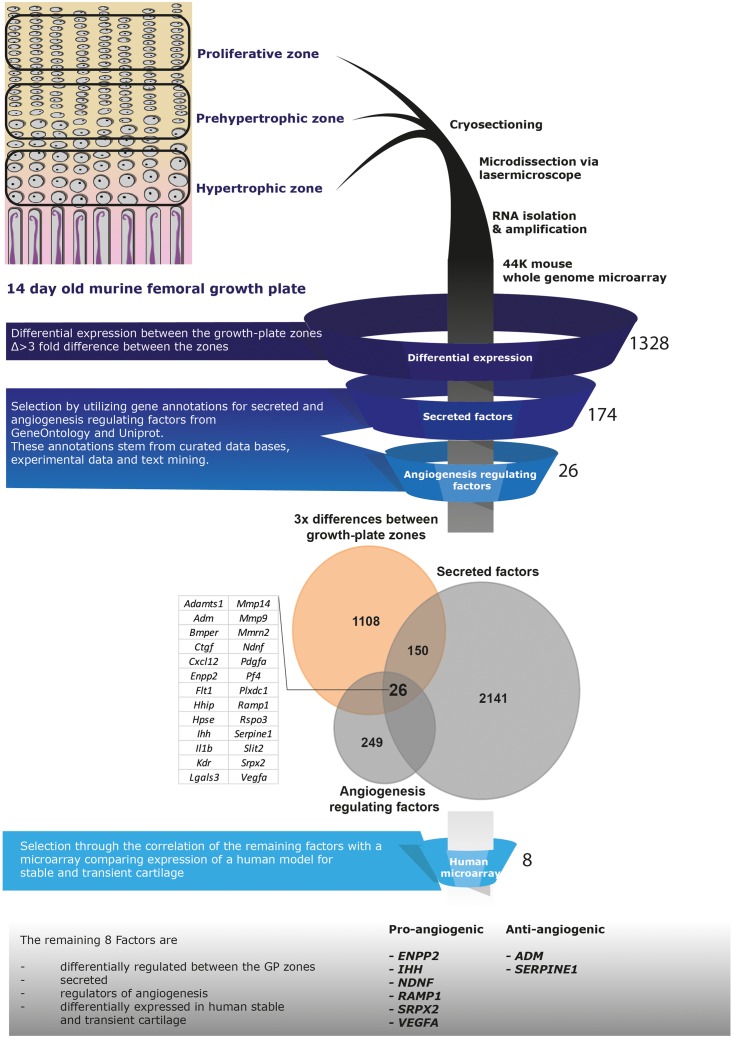
Selection pipeline for factors of interest. Diagram of the selection process of factors of interest starting from a growth plate microarray going through the selection of secreted, angiogenesis regulating factors via online resources ending at the comparison to a human dataset including neonatal cartilage and chondrogenically differentiated BMSC data. Twenty-six factors were identified being differentially expressed between the growth plate zones, secreted and regulate angiogenesis (Venn-diagram). From these, 8 factors appeared differentially expressed between human chondrogenically differentiated BMSC and neonatal articular cartilage.

To identify angiogenic factors in the secretome of hypertrophically differentiated chondrogenic cells we analyzed the expression of genes in data sets of growth plate cartilage, chondrogenic BMSC and neonatal cartilage. The different zones of the growth plate represent different phenotypical stages from cartilage to bone. We utilized microarray data comparing the three different zones (proliferative, pre-hypertrophic, and hypertrophic) of murine growth plate (Belluoccio et al., 2010) to determine which genes are differentially regulated in these zones. 1,328 genes showed a 3-fold difference in expression between two of the zones and among these 174 correspond to known secreted factors and 26 of these genes are described to regulate angiogenesis based according to online resource analysis (Uniprot, GeneOntology matrisome project database, Pubmed; Figure 4). We then compared the remaining 26 genes to a human microarray dataset that compared day 21 chondrogenically differentiated human BMSC pellets to neonatal articular cartilage (Somoza et al., 2018). Genes that were not differentially expressed in the human BMSC pellets and neonatal cartilage were discarded which led to the identification of eight genes: *ENPP2, IHH, NDNF, RAMP1, SPRX2, VEGFa, ADM, SERPIN E1* (Figure 4).

We then determined the gene expression levels of the final eight factors via qPCR, comparing our transient cartilage model (chondrogenically differentiated BMSC pellets) and a stable cartilage model of culture expanded articular chondrocytes differentiated as pellets following the same protocol used for BMSCs. *IHH* and *SERPINE1* were expressed significantly higher in BMSC-derived than in articular chondrocyte pellets (Table 1), confirming the data obtained from both microarrays. While the expression of *ENPP2, NDNF, SPRX2, VEGFa*, and *ADM*, showed no significant differences between transient and stable cartilage. *RAMP1* appeared to have a low expression in both the murine and human microarrays and was undetectable by PCR. This led us to select IHH and SERPINE1 as the most relevant factors for further follow-up.

**TABLE 1:** Selection of IHH and SERPINE1 via microarray and PCR analysis.

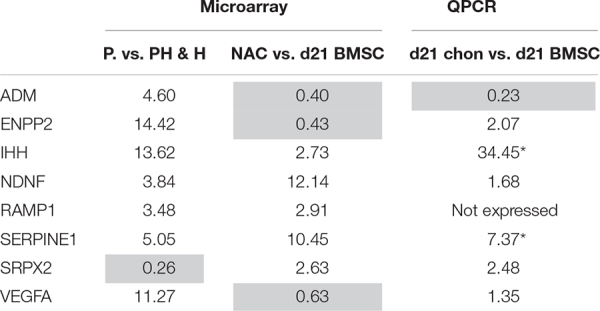

Expression of the 8 selected factors. (A) Expression pattern (increase or decrease) of the factors of interest comparing growth plate zones with neonatal articular cartilage (NAC) with chondrogenically differentiated BMSC and further with the gene expression pattern of the transient cartilage used in this study compared to a model of stable cartilage derived from 21 d differentiated chondrocytes (chon, chondrocyte derived pellet). *Significant difference in gene-expression analysis p = 0.001 tested with a Mann Whitney U-test. P, proliferative zone; PH, pre-hypertrophic zone; H, hypertrophic zone.

### Blocking IHH and Serpin E1 Increases Angiogenic Potential of Transient Cartilage Secretome

**FIGURE 5:**
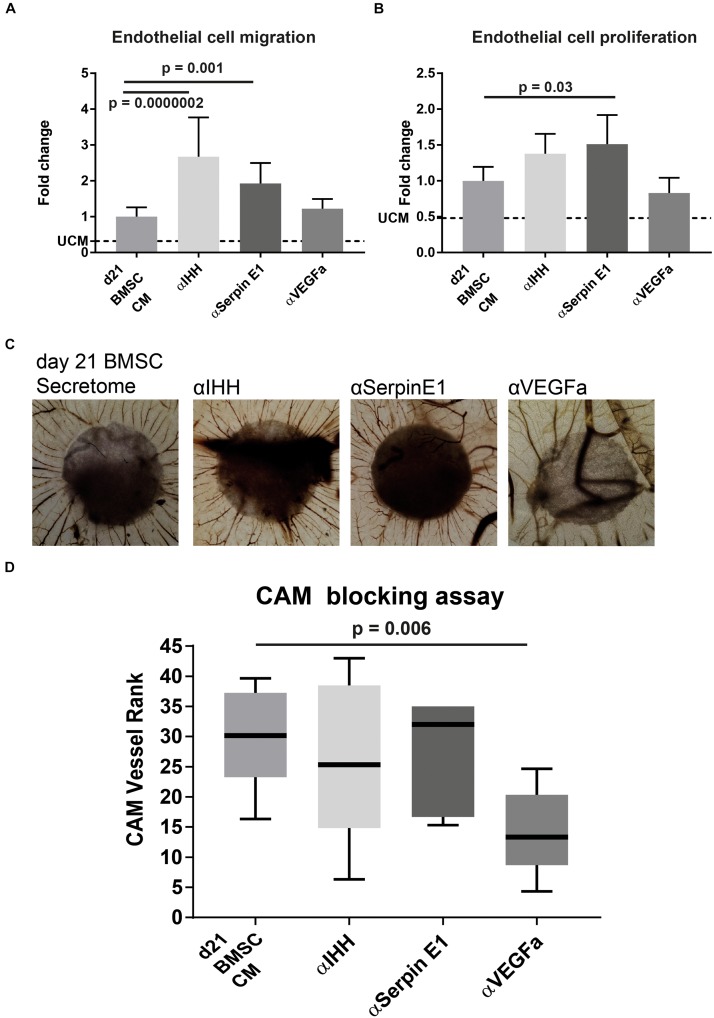
Blocking IHH or Serpin E1 increased angiogenic potential of CM of chondrogenically differentiated BMSC *in vitro* and showed no effect *in vivo* CAM assay. **(A)** Quantification of endothelial cell migration assay comparing the impact of pre-incubation of CM with blocking antibodies for factors of interest. Values are normalized to the BMSC-CM condition. UCM depicts the un-conditioned medium. **(B)** Quantification of endothelial cell proliferation assay comparing the impact of pre-incubation of CM with blocking antibodies for factors of interest. Values are normalized to the BMSC-CM condition. UCM depicts the un-conditioned medium. **(C)** Images of chick chorioallantoic membrane incubated for 72 h with CM (blocked for 30 min) soaked 5 mm filter disks. Filter circles were placed on a vessel free area on day 7 of the chick development. **(D)** Boxplot depicting the ranking of the angiogenic potential (lowest = 1 highest = 45: average of 3 independent observers: box = interquartile range, whiskers + 1–99%). *N* = 5 per condition. A & B were performed in two batches each pooling 3 donors; *n* = 3. Significance was determined by mixed-linear model with Bonferroni *post hoc* test.

To confirm the role of IHH and Serpin E1 present in BMSC CM on angiogenesis, we conducted the *in vitro* angiogenesis assays in the presence of neutralizing antibodies against IHH and Serpin E1. In addition, we used a neutralizing antibody against the major bioactive pro-angiogenic factor VEGF. In the endothelial migration assay, blocking Serpin E1 led to a significant increase in migration of endothelial cells of 1.9 (±0.6) (*p* = 0.001) fold and 2.7 (±1.1) (*p* = 0.0000002) fold when blocking IHH (Figure 5A). Interestingly, the angiogenic potential of the CM was not influenced by the presence of the VEGFa blocking antibody. Similar behavior was observed in the proliferation assay where blocking Serpin E1 led to 1.5 (±0.4) (*p* = 0.03) fold increase, blocking IHH increased endothelial cell proliferation by 1.4 (±0.3) fold, albeit not significant (*p* = 0.2), and no observed effect of the VEGFa blocking antibody (Figure 5B).

Blocking either IHH or Serpin E1 in the CAM assay did not increase the number of vessels or their directionality (Figures 5C,D). Blocking VEGFa showed a statistically significant (*p* = 0.006), reduction in the pro-angiogenic capacity of the chondrogenically differentiated BMSC secretome.

## Discussion

In this study, we identified soluble factors in BMSC-derived cartilage secretome that modulate angiogenesis. We first confirmed that the secretome of BMSC-derived cartilage is pro-angiogenic. Through a detailed analysis of multiple microarray datasets, Serpin E1 and IHH were identified as potential modulators of the angiogenic effect in our BMSC CM. Experiments where IHH and Serpin E1 were blocked demonstrated a further enhancement of the angiogenic potential of the chondrogenic BMSC CM. This indicated that IHH and Serpin E1 contribute as anti-angiogenic modulators to the process of angiogenesis. Despite the fact that the role of recombinant IHH and Serpin E1 in angiogenesis is already established in the literature (Isogai et al., 2001; Devy et al., 2002; Chinchilla et al., 2010; Wu et al., 2015), to our knowledge this is the first study where their presence and role have been identified in the context of the secretome of a transient human cartilage model.

Serpin E1 has been shown to be both pro (Devy et al., 2002; Wu et al., 2015) and anti-angiogenic (Isogai et al., 2001; Devy et al., 2002; Stefansson et al., 2003) under different conditions. This is dependent, for example, on the presence of fibronectin or vitronectin as well as on its own concentration. As Serpin E1 is part of the plasminogen activator system, it may further regulate angiogenesis through the inhibition of the activation of serine proteases. This effect has been studied in cancer and shows an upregulation of the plasminogen activator as well as Serpin E1. This may protect the fibrin-rich ECM to provide a scaffold for endothelial cell invasion (Pepper, 2001). This clear anti-angiogenic capacity might be explained by the previously described mechanism in which Serpin E1 in the presence of vitronectin prevents the interaction of Integrin αvβ3 with KDR (Isogai et al., 2001). The anti-angiogenic capacity of Serpin E1 is further supported by *in vivo* CAM data showing recombinant Serpin E1 preventing FGF2 induced angiogenesis (Stefansson et al., 2001). An additional possibility for Serpin E1 to influence angiogenesis is through the plasminogen system and its influence on fibrin which has been linked to angiogenesis during endochondral ossification (DeSimone and Reddi, 1992; Yuasa et al., 2015). By targeting Serpin E1 in a bone defect, fibrinolysis may increase depending on the presence of plasminogen activator and hence create an enhanced environment for vessel invasion and subsequent bone-repair (Yuasa et al., 2015).

IHH is known to be important in the transitioning from cartilage to bone through signaling in the pre-and hypertrophic zones in the growth plate (Chung et al., 2001). As a recombinant protein, it is slightly pro-angiogenic (Chinchilla et al., 2010; Chapouly et al., 2019) while during endochondral ossification, it has been described in a murine *in vivo* model to negatively impact vessel expansion and persistence (Colnot et al., 2005). Blocking the function of IHH in the context of our transient cartilage model resulted in a clear increase in the migration and a slight increase in the proliferation of endothelial cells, supporting the anti-angiogenic effect of IHH. This provides an *in vitro* confirmation the results shown by Colnot et al. (2005) where an anti-angiogenic effect of IHH using a conditional IHH knock-out mouse model was observed. In addition to the role of IHH in the PTHrP feedback loop and in osteoblast formation (Ohba, 2016), this suggests a role in modulating angiogenesis during endochondral ossification.

We demonstrated the pro-angiogenic effect through different *in vitro* as well as *in vivo* angiogenesis assays. Our data is at odds with the findings of Bara et al. (2014) who used chondrogenically differentiated equine BMSC pellet CM (conditioned for 96 h) on a Matrigel-tube formation assay with HUVECs to conclude the secretomes anti-angiogenic capacity. The difference observed might be attributed to differences in cell source, generation of CM or choice of assays. For our study, we used human endothelial cells for the *in vitro* assays and to evaluate *in vivo* angiogenesis, we have performed the CAM assay. Taken together, there is a strong indication that the CM is pro-angiogenic while the Matrigel tube-formation assay, focuses on the network organization of cells which is not indicative for angiogenesis (Simons et al., 2015). The CAM assay comes with the limitation that human secreted factors are used to induce avian blood vessel formation. Furthermore, this assay provides a rather qualitative assessment of the angiogenesis modulating capacity of a factor. This might have (partly) caused the absence of an observed effect of blocking Serpin E1 or IHH in this assay. It is also possible that further improvement of the already pro-angiogenic signal might have not been achievable (i.e., we reached the limits of possible blood vessel formation in the CAM using our CM). We did, however, observe a significant reduction of the pro-angiogenic effect of the secretome when using a VEGFa blocking antibody, indicating that modulation of angiogenesis induced by CM from BMSC in the CAM assay is possible. Interestingly, the inhibition of angiogenesis using VEGFa blocking antibodies was absent in the *in vitro* angiogenesis assays. The blocking antibody was checked for its function against recombinant VEGF where a diminished pro-angiogenic effect *in vitro* was observed. This suggests that VEGFa in the CM does not primarily act on endothelial cell migration or proliferation but could influence other sub-processes of angiogenesis (Shin et al., 2016). Furthermore, it was previously shown that the BMSC derived cartilage secretome contains VEGF (Farrell et al., 2009).

Initially, we expected to identify pro-angiogenic proteins by selecting factors upregulated in the pre-hypertrophic and hypertrophic zone of the growth plate and in transient cartilage generated by BMSCs. However, our two top hits were anti-angiogenic in our assays. In order to identify possible factors that are not categorized in gene ontology tools as “angiogenesis regulating” we removed this criterion from our search and ran the *in silico* analysis again. The previously described selection pipeline was repeated for both the growth plate and the human BMSC-derived cartilage microarray datasets, excluding the extra criterion of “known angiogenic factors.” Comparing the expression profiles, the lists of differentially expressed secreted factors lead to the identification of 16 genes encoding for secreted factors: *MMP10, HPSE, TOR3A, PENK, SRGN, LOX, IGFBP3, MMP11, COL10A1, IGFBP5, COL5A2, GAS6, POSTN, F13A1, SPON2, EPDR1*. These were uniquely upregulated in transient cartilage generated by BMSCs compared to neonatal articular cartilage and in the pre-/hypertrophic zone of the GP compared to the proliferative zone. One or more of these 16 factors might hold the explanation for the partially VEGFa independent pro-angiogenic potential shown by the CM.

This study focused on the angiogenic potential of BMSC-derived cartilaginous constructs, which are known to undergo endochondral ossification after subcutaneous implantation (Scotti et al., 2010; Farrell et al., 2011; Staines et al., 2013; Thompson et al., 2015). This process is dependent on the invasion of blood vessels, likely due to the pro-angiogenic capacity of the secretome (Gerber et al., 1999; Yang et al., 2012; Grosso et al., 2017). The set-up of our study had some limitations. Since we have pooled conditioned medium of three donors, not considering the sex of the donor we cannot exclude sex-dependent differences. Moreover, BMSC were obtained from patient with osteoarthritis. Although chondrogenically differentiated BMSC derived from healthy and OA donors have both demonstrated to undergo endochondral ossification when implanted *in vivo* (Yang et al., 2015) – which was also demonstrated in other mammals (Janicki et al., 2011; Sheehy et al., 2015) – we cannot fully exclude that the disease might have affected the angiogenic potential of the cells. Finally, it is well known that undifferentiated BMSC have a pro-angiogenic capability (Du et al., 2016) as well. Whether or not the factors secreted by chondrogenically differentiated BMSC are similar or different to undifferentiated BMSC remains to be investigated.

The work presented in this article shows that the secretome from chondrogenically differentiated human bone marrow derived BMSCs is pro-angiogenic. We further show that IHH and Serpin E1 are uniquely upregulated factors during chondrogenesis and endochondral ossification that act in an anti-angiogenic fashion in this context. This might be useful in articular cartilage repair approaches by taking advantage of the anti-angiogenic capacity demonstrated by IHH & Serpin E1. Furthermore, identification of a unique pro-angiogenic secreted factor to target would also offer potential solutions for the generation of stable articular cartilage. On the other hand, chondrogenic differentiation of adult human BMSCs provides a pro-angiogenic secretome that may be used to enhance bone regeneration and repair, which could be further enhanced by reducing the effect of IHH & Serpin E1.

## Data Availability Statement

The human datasets for this article is available in the GEO-Microarray database (GSE140861). The murine growth-plate microarray data is available in the GEO-Microarray database (GSE144362).

## Ethics Statement

The studies involving human participants were reviewed and approved by the Medical Ethical Committee Erasmus MC. The patients/participants provided their written informed consent to participate in this study.

## Author Contributions

YN was involved in the conception and design, collection of data, data analysis, interpretation, and manuscript drafting and editing. EF was involved in the conception, data analysis, interpretation, manuscript drafting, and editing. WK was involved in the collection of data and editing of materials and methods. RS and AC was involved in the data analysis and interpretation of the human microarray dataset, and manuscript editing. BB was involved in the data analysis and interpretation of the murine microarray dataset, and manuscript editing. GO was involved in the conception and design, data analysis and interpretation, and manuscript drafting and editing. All authors approved the final version of the manuscript.

## Conflict of Interest

The authors declare that the research was conducted in the absence of any commercial or financial relationships that could be construed as a potential conflict of interest.

## Footnotes

1 http://www.funrich.org/
